# An investigation of IL-8 degradation in response to PA401 compared to hypertonic saline in bronchoalveolar lavage fluid of Cystic Fibrosis patients

**DOI:** 10.1186/1753-6561-9-S1-A12

**Published:** 2015-01-14

**Authors:** H Kerr, O’Reilly, OJ McElvaney, DA Bergin

**Affiliations:** 1 Royal College of Surgeons in Ireland, 123 St. Stephen’s Green, Dublin, Ireland; 2 Department of Medicine, Beaumont Hospital, Dublin, Ireland

## Background

The lung pathogenesis of cystic fibrosis (CF) involves inflammation, airway obstruction and an increased incidence of pulmonary infections. Increased levels of proinflammatory cytokines and chemokines such as interleukin-8 (IL-8) play a pivotal role in sustaining the cycle of inflammation in the CF lung. Glycosaminoglycans (GAGs) possess the ability to bind IL-8 providing protection from proteolytic degradation and maintaining it in an active state leading to sustained neutrophil chemotaxis. It has been shown that hypertonic saline (HTS) disrupts GAG:IL-8 complexes, thus rendering IL-8 susceptible to proteolysis thereby reducing neutrophil chemotaxis. The recombinant IL-8 decoy (PA401) binds glycans with higher affinity (x 40) than native IL-8. In this study, we compared the ability of PA401 and HTS to disrupt IL-8:GAG complexes in CF BALF.

## Methods

IL-8 concentration in CF BALF was determined following exposure to PA401 or HTS by ELISA. PA401 degradation in CF BALF (± protease inhibitors) was examined using gradient SDS-PAGE and Western Blot analysis employing a primary antibody specific for the PA401 decoy (MAB8A12).

## Results

Individual CF BALF samples (n=7) displayed a high level of variability with regard to IL-8 concentration and response to PA401 or HTS. Exposure of pooled CF BAL to increasing concentrations of PA401 lead to a significant decrease in the level of detectable IL-8 (p<0.05) and neutrophil chemotactic efficiency (30 %, p<0.05). Significantly reduced levels of IL-8 (p<0.05) were detected following incubation with PA401 for 4 hr in 6/7 individuals with CF when compared to a PBS control. The level of IL-8 present in BALF following incubation with PA401 was significantly reduced compared to HTS (p<0.05) in 2/3 CF patients. Western Blot analysis indicated that serine proteases (inhibited by alpha-1 antitrypsin, PMSF and pefabloc) play a major role in degrading PA401.

## Conclusions

The reduced levels of IL-8 in BALF samples treated with PA401 revealed that PA401 is likely to be effective in disrupting IL-8:GAG complexes in the CF lung rendering IL-8 susceptible to proteolysis and thus may be seen as a therapeutic target in the treatment of CF. Further benefits of PA401 are evident as the decoy did not accumulate in CF samples and post IL-8 clearance, it too was degraded by serine and metalloproteases. Clinical application of an IL-8 decoy may serve to decrease the inflammatory burden in the CF lung *in vivo.*

